# Supplementary material for: Engineering of SauriCas9 with enhanced specificity
Source: Mol Ther Nucleic Acids. 2025 Jan 17;36(1):102455. doi: 10.1016/j.omtn.2025.102455 (PMC11869866; doi:10.1016/j.omtn.2025.102455)
Supplement: Document S1. Figures S1–S3 [file mmc1.pdf]

OMTN, Volume *36*

Supplemental information

Engineering of SauriCas9

with enhanced specificity

Xiaoqi Zhang, Chen Tao, Miaomiao Li, Sufang Zhang, Puping Liang, Yan Huang, Huihui Liu, and Yongming Wang

TableS1 SauriCas9 and SauriCas9 variants

Table S2. Primers used in this study

Table S3. Target sites used in this study

Table S4. Data in this study

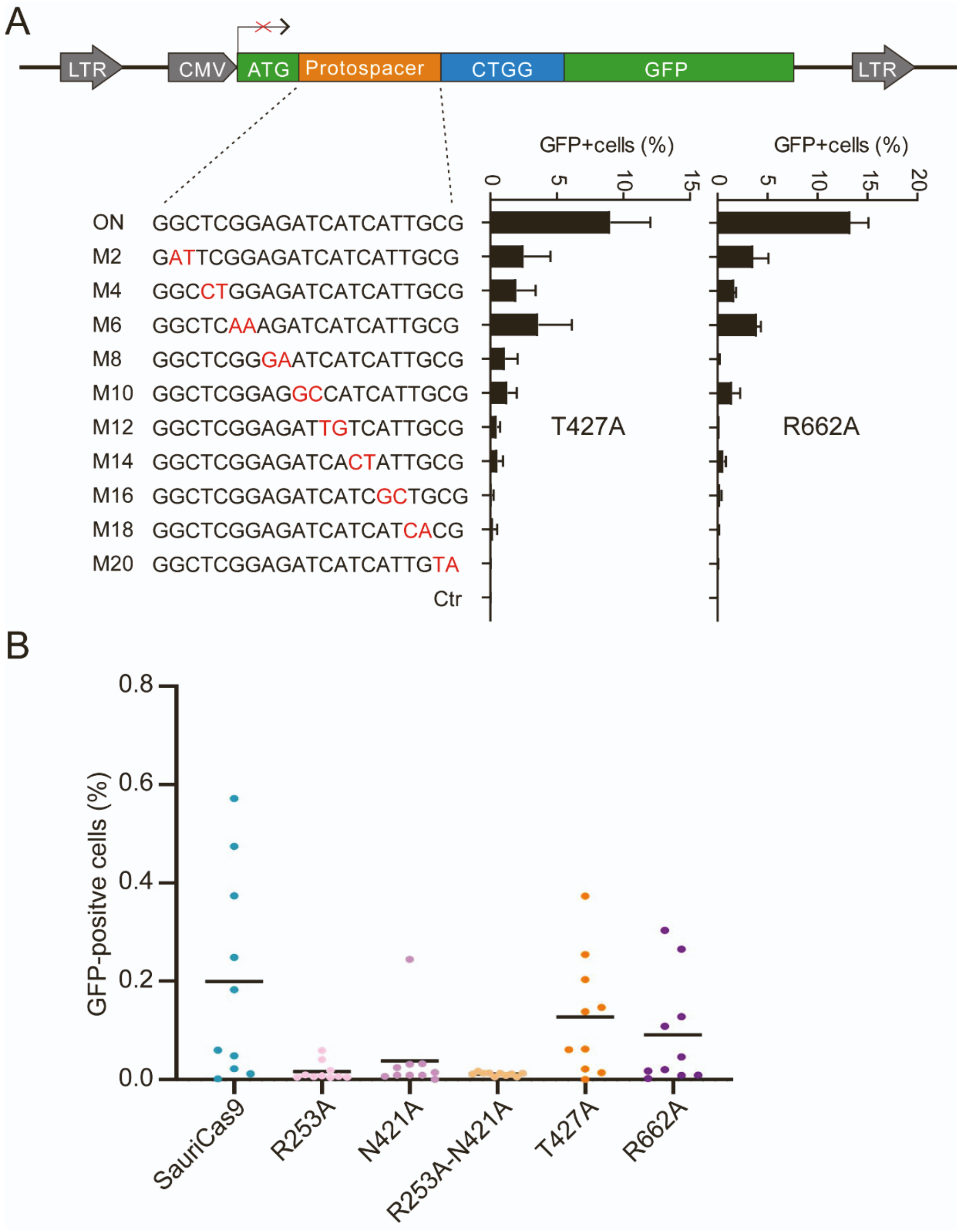

Fig. S1 Influence of mutations on SauriCas9 specificity. (A) Evaluation of Cas9 specificity using the GFP-activation assay. The top section illustrates the schematic of the GFP-activation reporter. A series of sgRNAs with dinucleotide mutations (depicted in red) is displayed below. The percentage of GFP-positive cells for each sgRNA is indicated on the right. The on-target sgRNA is included for reference. Ctr: reporter cells without Cas9-expressing plasmid transfection. **(B)** Comparison of editing efficiency at off-targets based on GFP-activation assay among six SauriCas9 variants in HEK293T cells. The editing efficiency at off-targets is normalized by on-target efficiency. Low editing efficiency indicates high specificity.

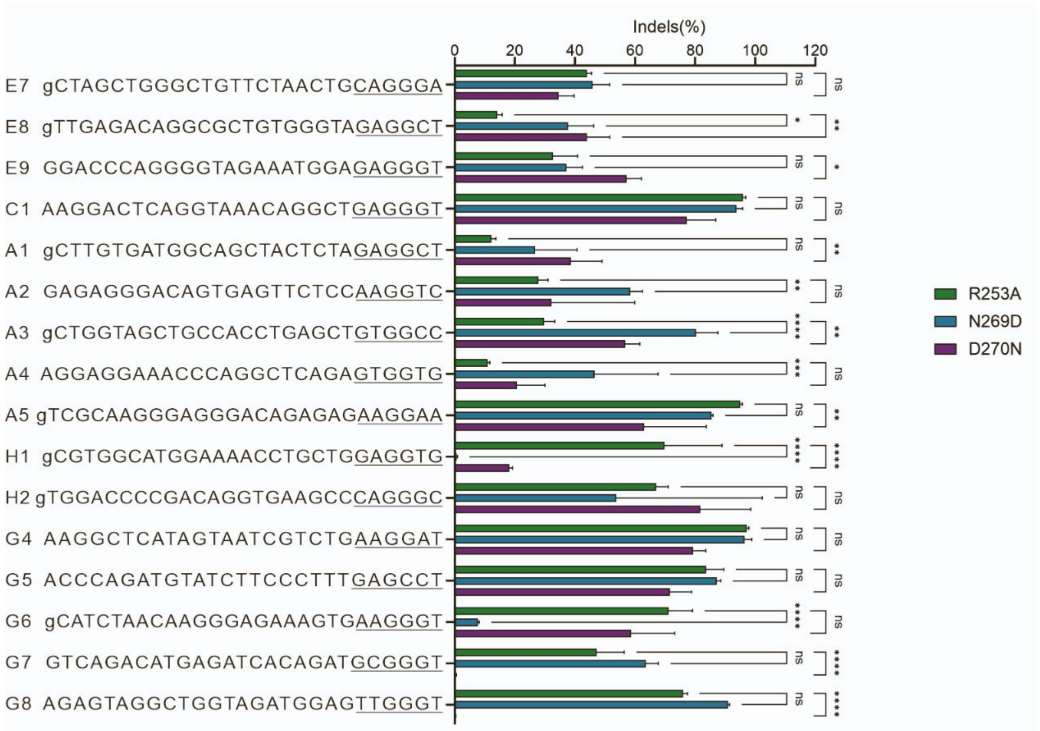

Fig. S2 Evaluation of the activity of three Cas9 variants at sixteen endogenous loci. The data is presented as mean ± SD (n = 3). Two-tailed, paired Student t-tests were utilized to determine statistical significance when comparing two groups, whereas analyses of variance (ANOVAs) were employed for comparisons involving three or more groups. A value of $P < 0.05$ was deemed statistically significant (*$P < 0.05$, **$P < 0.01$, ***$P < 0.001$).

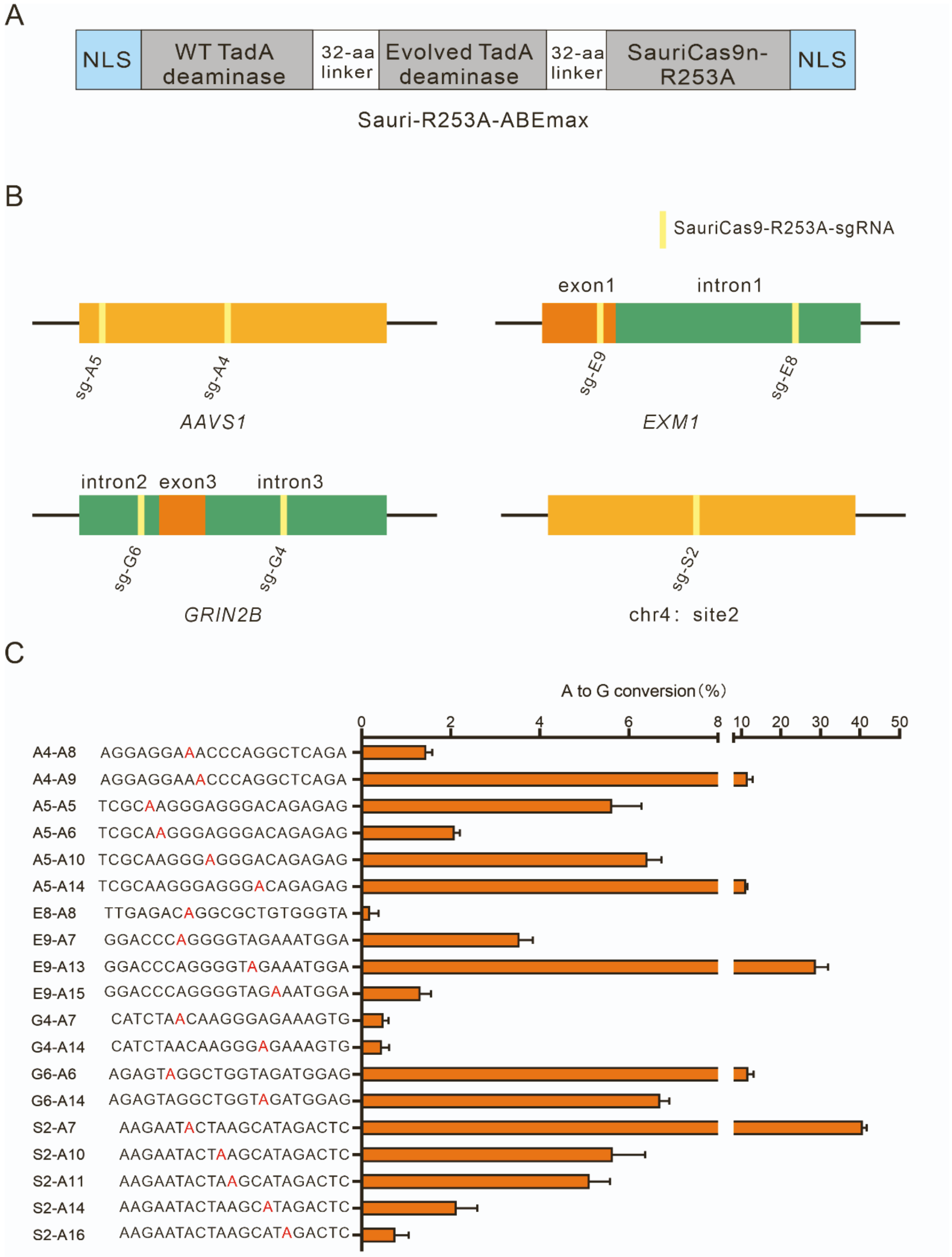

Fig. S3 Base editing with SauriCas9-R253A. (A) Schematic of the Sauri-R253A-ABEmax. **(B)** Schematic representation of sgRNA design targeting AAVS1, EXM1, GRIN2B and site2. Yellow boxes indicate the sgRNA targeting sites. **(C)** Sauri-R253A-ABEmax induces A-to-G conversions for a panel of 7 genomic loci (*n* = 3). Underlying data for all summary statistics can be found in Data. "A4-A8" means A at target A4 position 8. aa, amino acid; NLS, nuclear localization signal; Sauri-R253A-ABEmax, TadA-TadA*(involved TadA)–SauriCas9n-R253A; SauriCas9, Cas9 nuclease from *S. auricularis*; SauriCas9n, nickase form of SauriCas9.

References

1. Jinek, M., Chylinski, K., Fonfara, I., Hauer, M., Doudna, J.A., and Charpentier, E. (2012). A programmable dual-RNA-guided DNA endonuclease in adaptive bacterial immunity. Science *337*, 816-821. 10.1126/science.1225829.
2. Nishimasu, H., Cong, L., Yan, W.X., Ran, F.A., Zetsche, B., Li, Y., Kurabayashi, A., Ishitani, R., Zhang, F., and Nureki, O. (2015). Crystal Structure of Staphylococcus aureus Cas9. Cell *162*, 1113-1126. 10.1016/j.cell.2015.08.007.
3. Ran, F.A., Hsu, P.D., Wright, J., Agarwala, V., Scott, D.A., and Zhang, F. (2013). Genome engineering using the CRISPR-Cas9 system. Nature Protocols *8*, 2281-2308. 10.1038/nprot.2013.143.
4. Komor, A.C., Kim, Y.B., Packer, M.S., Zuris, J.A., and Liu, D.R. (2016). Programmable editing of a target base in genomic DNA without double-stranded DNA cleavage. Nature *533*, 420-424. 10.1038/nature17946.
5. Gaudelli, N.M., Komor, A.C., Rees, H.A., Packer, M.S., Badran, A.H., Bryson, D.I., and Liu, D.R. (2017). Programmable base editing of A*T to G*C in genomic DNA without DNA cleavage. Nature *551*, 464-471. 10.1038/nature24644.
6. Anzalone, A.V., Randolph, P.B., Davis, J.R., Sousa, A.A., Koblan, L.W., Levy, J.M., Chen, P.J., Wilson, C., Newby, G.A., Raguram, A., and Liu, D.R. (2019). Search-and-replace genome editing without double-strand breaks or donor DNA. Nature *576*, 149-157. 10.1038/s41586-019-1711-4.
7. Cong, L., Ran, F.A., Cox, D., Lin, S., Barretto, R., Habib, N., Hsu, P.D., Wu, X., Jiang, W., Marraffini, L.A., and Zhang, F. (2013). Multiplex genome engineering using CRISPR/Cas systems. Science *339*, 819-823. 10.1126/science.1231143.
8. Mali, P., Yang, L., Esvelt, K.M., Aach, J., Guell, M., DiCarlo, J.E., Norville, J.E., and Church, G.M. (2013). RNA-guided human genome engineering via Cas9. Science *339*, 823-826. 10.1126/science.1232033.
9. Ran, F.A., Cong, L., Yan, W.X., Scott, D.A., Gootenberg, J.S., Kriz, A.J., Zetsche, B., Shalem, O., Wu, X., Makarova, K.S., et al. (2015). In vivo genome editing using Staphylococcus aureus Cas9. Nature *520*, 186-191. 10.1038/nature14299.
10. Edraki, A., Mir, A., Ibraheim, R., Gainetdinov, I., Yoon, Y., Song, C.Q., Cao, Y., Gallant, J., Xue, W., Rivera-Perez, J.A., and Sontheimer, E.J. (2019). A Compact, High-Accuracy Cas9 with a Dinucleotide PAM for In Vivo Genome Editing. Mol Cell *73*, 714-726 e714. 10.1016/j.molcel.2018.12.003.
11. Kim, E., Koo, T., Park, S.W., Kim, D., Kim, K., Cho, H.Y., Song, D.W., Lee, K.J., Jung, M.H., Kim, S., et al. (2017). In vivo genome editing with a small Cas9 orthologue derived from Campylobacter jejuni. Nat Commun *8*, 14500. 10.1038/ncomms14500.
12. Hu, Z., Wang, S., Zhang, C., Gao, N., Li, M., Wang, D., Wang, D., Liu, D., Liu, H., Ong, S.G., et al. (2020). A compact Cas9 ortholog from Staphylococcus Auricularis (SauriCas9) expands the DNA targeting scope. PLoS Biol *18*, e3000686. 10.1371/journal.pbio.3000686.
13. Hu, Z., Zhang, C., Wang, S., Gao, S., Wei, J., Li, M., Hou, L., Mao, H., Wei, Y., Qi, T., et al. (2021). Discovery and engineering of small SlugCas9 with broad targeting range and high specificity and activity. Nucleic Acids Res *49*, 4008-4019. 10.1093/nar/gkab148.
14. Wang, S., Mao, H.L., Hou, L.H., Hu, Z.Y., Wang, Y., Qi, T., Tao, C., Yang, Y., Zhang, C.D., Li, M.M., et al. (2021). Compact SchCas9 Recognizes the Simple NNGR PAM. Advanced

Science. Artn 2104789
10.1002/Advs.202104789.
15. Wei, J.J., Hou, L.H., Liu, J.T., Wang, Z.W., Gao, S.Q., Qi, T., Gao, S., Sun, S.A., and Wang, Y.M. (2022). Closely related type II-C Cas9 orthologs recognize diverse PAMs. Elife *11*. ARTN e77825
10.7554/eLife.77825.
16. Wang, S., Tao, C., Mao, H.L., Hou, L.H., Wang, Y., Qi, T., Yang, Y., Ong, S.G., Hu, S.J., Chai, R.J., and Wang, Y.M. (2022). Identification of SaCas9 orthologs containing a conserved serine residue that determines simple NNGG PAM recognition. Plos Biology *20*. ARTN e3001897
10.1371/journal.pbio.3001897.
17. Gao, S.Q., Wang, Y., Qi, T., Wei, J.J., Hu, Z.Y., Liu, J.T., Sun, S.A., Liu, H.H., and Wang, Y.M. (2023). Genome editing with natural and engineered CjCas9 orthologs. Molecular Therapy *31*, 1177-1187. 10.1016/j.ymthe.2023.01.029.
18. Liu, J.J., Orlova, N., Oakes, B.L., Ma, E., Spinner, H.B., Baney, K.L.M., Chuck, J., Tan, D., Knott, G.J., Harrington, L.B., et al. (2019). CasX enzymes comprise a distinct family of RNA-guided genome editors. Nature *566*, 218-223. 10.1038/s41586-019-0908-x.
19. Kim, D.Y., Lee, J.M., Moon, S.B., Chin, H.J., Park, S., Lim, Y., Kim, D., Koo, T., Ko, J.H., and Kim, Y.S. (2022). Efficient CRISPR editing with a hypercompact Cas12f1 and engineered guide RNAs delivered by adeno-associated virus. Nature Biotechnology *40*, 94-+. 10.1038/s41587-021-01009-z.
20. Xu, X.S., Chemparathy, A., Zeng, L.P., Kempton, H.R., Shang, S., Nakamura, M., and Qi, L.S. (2021). Engineered miniature CRISPR-Cas system for mammalian genome regulation and editing. Molecular Cell *81*, 4333-+. 10.1016/j.molcel.2021.08.008.
21. Wu, Z.W., Zhang, Y.F., Yu, H.P., Pan, D., Wang, Y.J., Wang, Y.N., Li, F., Liu, C., Nan, H., Chen, W.Z., and Ji, Q.J. (2021). Programmed genome editing by a miniature CRISPR-Cas12f nuclease. Nature Chemical Biology *17*, 1132-1138. 10.1038/s41589-021-00868-6.
22. Pausch, P., Al-Shayeb, B., Bisom-Rapp, E., Tsuchida, C.A., Li, Z., Cress, B.F., Knott, G.J., Jacobsen, S.E., Banfield, J.F., and Doudna, J.A. (2020). CRISPR-Cas Phi from huge phages is a hypercompact genome editor. Science *369*, 333-+. 10.1126/science.abb1400.
23. Wang, Y., Qi, T., Liu, J.T., Yang, Y., Wang, Z.W., Wang, Y., Wang, T.Y., Li, M.M., Li, M.Q., Lu, D.R., et al. (2023). A highly specific CRISPR-Cas12j nuclease enables allele- specific genome editing. Science Advances *9*. ARTN eabo6405
10.1126/sciadv.abo6405.
24. Slaymaker, I.M., Gao, L., Zetsche, B., Scott, D.A., Yan, W.X., and Zhang, F. (2016). Rationally engineered Cas9 nucleases with improved specificity. Science *351*, 84-88. 10.1126/science.aad5227.
25. Tan, Y., Chu, A.H.Y., Bao, S., Hoang, D.A., Kebede, F.T., Xiong, W., Ji, M., Shi, J., and Zheng, Z. (2019). Rationally engineered Staphylococcus aureus Cas9 nucleases with high genome-wide specificity. Proc Natl Acad Sci U S A *116*, 20969-20976. 10.1073/pnas.1906843116.
26. Lee, J.K., Jeong, E., Lee, J., Jung, M., Shin, E., Kim, Y.H., Lee, K., Jung, I., Kim, D., Kim, S., and Kim, J.S. (2018). Directed evolution of CRISPR-Cas9 to increase its specificity. Nature Communications *9*. ARTN 3048

10.1038/s41467-018-05477-x.

27. Tsai, S.Q., Zheng, Z., Nguyen, N.T., Liebers, M., Topkar, V.V., Thapar, V., Wyvekens, N., Khayter, C., Iafrate, A.J., Le, L.P., et al. (2015). GUIDE-seq enables genome-wide profiling of off-target cleavage by CRISPR-Cas nucleases. Nat Biotechnol *33*, 187-197. 10.1038/nbt.3117.
28. Wei, N., Shang, L., Liu, J., Wang, M., Liu, Y., Zhu, C., Fei, C., Zhang, L., Yang, F., and Gu, F. (2023). Engineered Staphylococcus auricularis Cas9 with high-fidelity. FASEB J *37*, e23060. 10.1096/fj.202202132RR.
29. Liu, X., Zhang, Y., Cheng, C., Cheng, A.W., Zhang, X., Li, N., Xia, C., Wei, X., Liu, X., and Wang, H. (2017). CRISPR-Cas9-mediated multiplex gene editing in CAR-T cells. Cell Res *27*, 154-157. 10.1038/cr.2016.142.
30. Riolobos, L., Hirata, R.K., Turtle, C.J., Wang, P.R., Gornalusse, G.G., Zavajlevski, M., Riddell, S.R., and Russell, D.W. (2013). HLA engineering of human pluripotent stem cells. Mol Ther *21*, 1232-1241. 10.1038/mt.2013.59.
31. Kamphorst, A.O., and Ahmed, R. (2013). Manipulating the PD-1 pathway to improve immunity. Curr Opin Immunol *25*, 381-388. 10.1016/j.coi.2013.03.003.
32. Knauer, C., Haltern, H., Schoger, E., Kugler, S., Roos, L., Zelarayan, L.C., Hasenfuss, G., Zimmermann, W.H., Wollnik, B., and Cyganek, L. (2024). Preclinical evaluation of CRISPR-based therapies for Noonan syndrome caused by deep-intronic LZTR1 variants. Mol Ther Nucleic Acids *35*, 102123. 10.1016/j.omtn.2024.102123.
33. Davis, J.R., Wang, X., Witte, I.P., Huang, T.P., Levy, J.M., Raguram, A., Banskota, S., Seidah, N.G., Musunuru, K., and Liu, D.R. (2022). Efficient in vivo base editing via single adeno-associated viruses with size-optimized genomes encoding compact adenine base editors. Nat Biomed Eng *6*, 1272-1283. 10.1038/s41551-022-00911-4.
34. Yang, L., Liu, Z., Sun, J., Chen, Z., Gao, F., and Guo, Y. (2024). Adenine base editor-based correction of the cardiac pathogenic Lmna c.1621C > T mutation in murine hearts. J Cell Mol Med *28*, e18145. 10.1111/jcmm.18145.
35. Crooks, G.E., Hon, G., Chandonia, J.M., and Brenner, S.E. (2004). WebLogo: a sequence logo generator. Genome Res *14*, 1188-1190. 10.1101/gr.849004.
