# Supplementary material for: Engineering of SauriCas9 with enhanced specificity
Source: Mol Ther Nucleic Acids. 2025 Jan 17;36(1):102455. doi: 10.1016/j.omtn.2025.102455 (PMC11869866; doi:10.1016/j.omtn.2025.102455)
Supplement: Document S2. Article plus supplemental information [file mmc6.pdf]

Molecular Therapy
Nucleic Acids
Original Article

Engineering of SauriCas9 with enhanced specificity

Xiaoqi Zhang,[1,6] Chen Tao,[1,6] Miaomiao Li,[1] Sufang Zhang,[2] Puping Liang,[3] Yan Huang,[4] Huihui Liu,[5] and Yongming Wang[1,4]

[1]Center for Medical Research and Innovation, Shanghai Pudong Hospital, Fudan University Pudong Medical Center, School of Life Sciences, Fudan University, Shanghai 200438, China; [2]Key Laboratory of Forest Protection of National Forestry and Grassland Administration, Ecology and Nature Conservation Institute, Chinese Academy of Forestry, Beijing 100091, China; [3]State Key Laboratory of Biocontrol, MOE Key Laboratory of Gene Function and Regulation and Guangzhou Key Laboratory of Healthy Aging Research, School of Life Sciences, Sun Yat-sen University, Guangzhou 510275, China; [4]State Key Laboratory of Genetic Engineering, Shanghai Engineering Research Center of Industrial Microorganisms, School of Life Science, Fudan University, Shanghai 200433, China; [5]National Permanent Scientific Research Base for Warm Temperate Zone Forestry of Jiulong Mountain in Beijing, Experimental Center of Forestry in North China, CAF, Beijing 102300, China

SauriCas9 is a compact Cas9 nuclease showing promise for *in vivo* therapeutic applications. However, concerns about off-target effects necessitated improvements in specificity. We addressed this by introducing mutations to eliminate polar contacts between Cas9 and the target DNA, resulting in the SauriCas9-R253A variant with enhanced specificity. To validate its efficacy, we employed SauriCas9-R253A to disrupt three genes (B2M, TRAC, and PDCD1), a strategy integral to the development of allogeneic chimeric antigen receptor T cell (CAR-T) therapies. Our results demonstrated that the most efficient single-guide RNAs for SauriCas9-R253A exhibited comparable activity to SpCas9 and showed no detectable off-target effects in the disruption of these genes, highlighting its therapeutic potential.

INTRODUCTION

RNA-guided CRISPR-Cas9 is a microbial adaptive immune system that has been extensively utilized for genome editing. This system comprises three crucial components: a Cas9 nuclease, a CRISPR RNA (crRNA), and a *trans*-activating crRNA (tracrRNA).[1] Together, they assemble into a ribonucleoprotein complex in which the crRNA specifies the target DNA by pairing with the target sequence, while the tracrRNA serves as a binding scaffold for the Cas9 nuclease.[2] The crRNA and tracrRNA can be fused together via a short linker to form a single-guide RNA (sgRNA).[1] Once the Cas9-sgRNA complex recognizes a target DNA, Cas9 initiates a cleavage of the DNA, generating a double-stranded DNA break (DSB). The DSB is repaired by either the non-homologous end joining or the homology-directed repair pathway, resulting in modifications to the genomic DNA.[3] In addition to conventional genome editing, the CRISPR-Cas9 system has been engineered for base editing[4,5] and prime editing.[6]

Several CRISPR-Cas9 nucleases derived from different microorganisms have been employed for genome editing. Among them, SpCas9 is the most widely used due to its high activity and simple protospacer-adjacent motif (PAM) requirements.[7,8] However, SpCas9 possesses a large genome size of approximately 4.1 kb, which, when combined with the sgRNA, exceeds the packaging capability of a single adeno-associated virus (AAV). As a response to this challenge, researchers have developed several compact CRISPR-Cas9 systems, including SaCas9,[9] Nme2Cas9,[10] CjCas9,[11] SauriCas9,[12] SlugCas9,[13] SchCas9,[14] Nsp2Cas9,[15] SpeCas9,[16] and Hsp1Cas9.[17] These compact systems can be efficiently delivered using a single AAV vector for *in vivo* genome editing. In addition, several compact CRISPR-Cas12 systems, including CasX,[18] Cas12f1,[19–21] and Cas12j,[22,23] have been used for genome editing.

Addressing off-target effects is a significant concern in genome editing. CRISPR-Cas9 systems can tolerate some mismatches between the crRNA and the target DNA, leading to unintended DNA cleavage at sequences resembling the intended target.[24] In the realm of research, off-target effects can complicate result interpretation, while in clinical applications, they may disrupt crucial coding regions, potentially causing genotoxic effects such as cancer. To enhance the specificity of CRISPR-Cas9, both rational design[24,25] and molecular-directed evolution approaches[26] have been employed. We previously developed a compact SauriCas9 with high activity and a simple dinucleotide PAM requirement.[12]

Received 12 May 2024; accepted 14 January 2025;
https://doi.org/10.1016/j.omtn.2025.102455.

[6]These authors contributed equally

Correspondence: Yan Huang, State Key Laboratory of Genetic Engineering, Shanghai Engineering Research Center of Industrial Microorganisms, School of Life Science, Fudan University, Shanghai 200433, China.
E-mail: huangyan@fudan.edu.cn
Correspondence: Huihui Liu, National Permanent Scientific Research Base for Warm Temperate Zone Forestry of Jiulong Mountain in Beijing, Experimental Center of Forestry in North China, CAF, Beijing 102300, China.
E-mail: liuhuihui800@caf.ac.cn
Correspondence: Yongming Wang, Center for Medical Research and Innovation, Shanghai Pudong Hospital, Fudan University Pudong Medical Center, School of Life Sciences, Fudan University, Shanghai 200438, China.
E-mail: ymw@fudan.edu.cn

Published by Elsevier Inc. on behalf of The American Society of Gene and Cell Therapy.
This is an open access article under the CC BY license (http://creativecommons.org/licenses/by/4.0/).

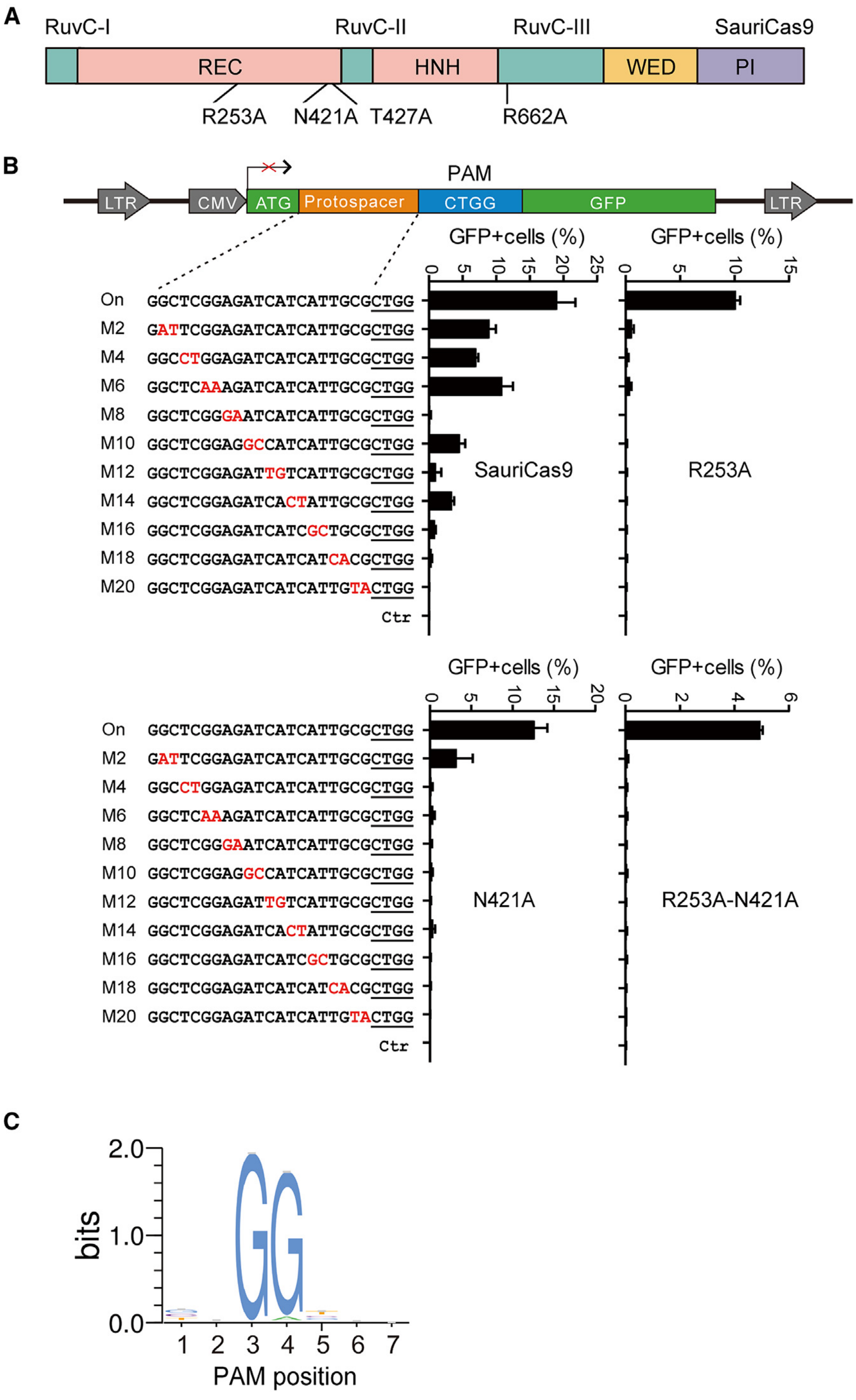

Figure 1. Influence of mutations on SauriCas9 specificity

(A) Schematic of the SauriCas9 structure highlighting the positions of four mutations. The sequence alignment of SaCas9 and SauriCas9 is shown below. (B) Evaluation of Cas9 specificity using the GFP-activation assay. The top section illustrates the schematic of the GFP-activation reporter, wherein the GFP expression is interrupted by the insertion of a target sequence (protospacer + PAM) between the ATG start codon and the coding sequence. A series of sgRNAs with dinucleotide mutations (depicted in red) is displayed below. The percentage of GFP^+ cells for each sgRNA is indicated at right. The on-target sgRNA is included for reference. Ctr, reporter cells without Cas9-expressing plasmid transfection. (C) PAM sequence analysis for SauriCas9-R253A. WebLogo is generated from deep sequencing data.

However, this system exhibited moderate specificity. In this study, we engineered SauriCas9 for improved specificity. In addition, we showed that SauriCas9 can efficiently disrupt β-2 microglobulin (*B2M*), programmed cell death 1 (*PDCD1*), and T cell receptor α constant (*TRAC*) genes with undetectable off-target effects.

RESULTS

A previous study identified four amino acid residues (R245, N413, N419, and R654) forming polar contacts with the target DNA strand by analyzing the crystal structure of the SaCas9/sgRNA-target DNA complex.[25] The replacement of these amino acid residues with alanine improved SaCas9 specificity.[25] Given the 62.4% sequence identity between SauriCas9 and SaCas9,[12] we sought to examine whether the corresponding residues could enhance SauriCas9 specificity. Through pairwise alignment, we identified the corresponding residues (R253, N421, T427, and R662) on SauriCas9 (Figure 1A). Each residue was replaced with alanine, and its impact on specificity was tested using a GFP-activation assay.[12] In this assay, a protospacer followed by a CTGG PAM was inserted between the ATG start codon and GFP coding sequence, inducing a frameshift mutation (Figure 1B). The reporter was stably integrated into the HEK293T cell genome via lentivirus. If a Cas9-sgRNA complex edits the protospacer and induces indels, then a portion of reporters will restore GFP expression.

We designed a set of off-target sgRNAs featuring dinucleotide mismatches targeting the protospacer. The efficiency of these sgRNAs is reflected by the percentage of GFP^+ cells. Cas9 specificity is determined by normalizing the efficiency of off-target sgRNAs against that of the on-target sgRNA. Three days post-transfection of sgRNA- and Cas9-expressing plasmids, GFP^+ cells were quantified using fluorescence-activated cell sorting. Wild-type SauriCas9 induced a 19.1% GFP^+ cell population, signifying efficient genome editing. The

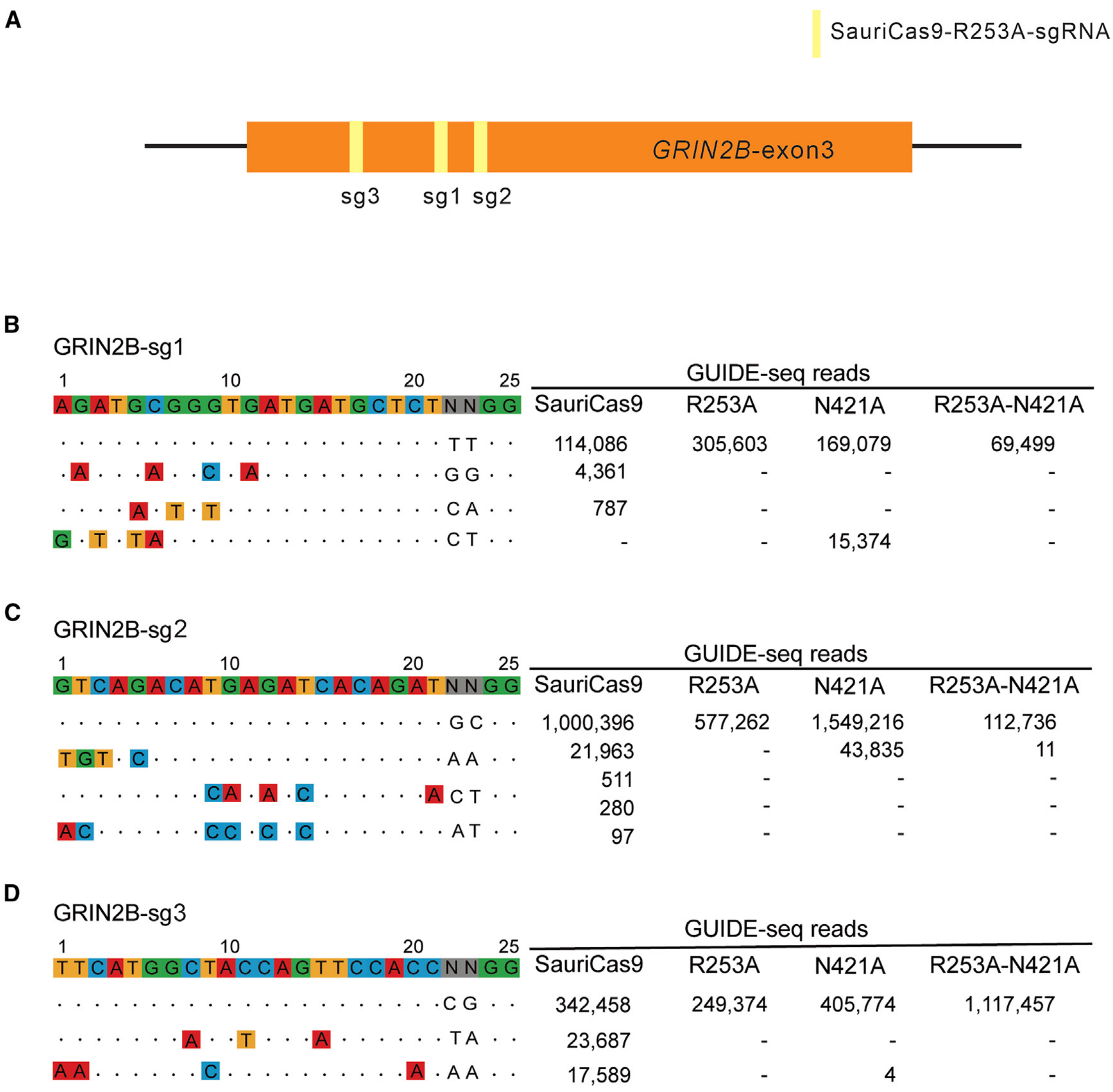

Figure 2. Evaluation of specificity for each SauriCas9 variant using the GUIDE-seq assay

(A) Schematic of the *GRIN2B* exon 3. Yellow boxes indicate the sgRNA targeting sites. (B–D) GUIDE-seq analysis of genome-wide off-target effects for three sgRNAs targeting the GRIN2B gene. The on-target sequences are presented at the top, while mismatches compared with the on-target sequence are depicted below. The read numbers for both on- and off-targets are provided at right.

sgRNAs with mismatches also led to up to 10.9% GFP^+ cells (Figure 1B), aligning with our previous findings. Intriguingly, all four mutations reduced off-target effects, with R253A and N421A exhibiting more substantial reductions (Figures 1B and S1A). Introducing double mutations (R253A and N421A) into SauriCas9 further reduced off-target effects. The specificity of six SauriCas9 variants ranked as R253A-N421A > R253A > N421A > R662A > T427A > SauriCas9 (Figure S1B). To investigate whether the mutations affect SauriCas9 PAM recognition, we employed a previously established GFP-activation assay[12] to assess the recognition pattern of the SauriCas9-R253A PAM. The results revealed that the R253A mutation did not alter SauriCas9 PAM preference (Figure 1C). Subsequent investigations focused on the Cas9 variants with R253A, N421A, and R253A + N421A mutations.

Next, we investigated the genome-wide off-target effects of SauriCas9, R253A, N421A, and R253A + N421A using the genome-wide, unbiased identification of DSBs enabled by sequencing (GUIDE-seq) assay[27] with three sgRNAs targeting the *GRIN2B* gene (Figure 2A). Upon transfection of the Cas9 variants, sgRNA plasmid, and GUIDE-seq oligos, we generated libraries for deep sequencing. The sequencing results and subsequent analysis demonstrated on-target cleavage for all Cas9 variants, evident from the elevated GUIDE-seq read counts (Figures 2B–2D). SauriCas9, N421A, and

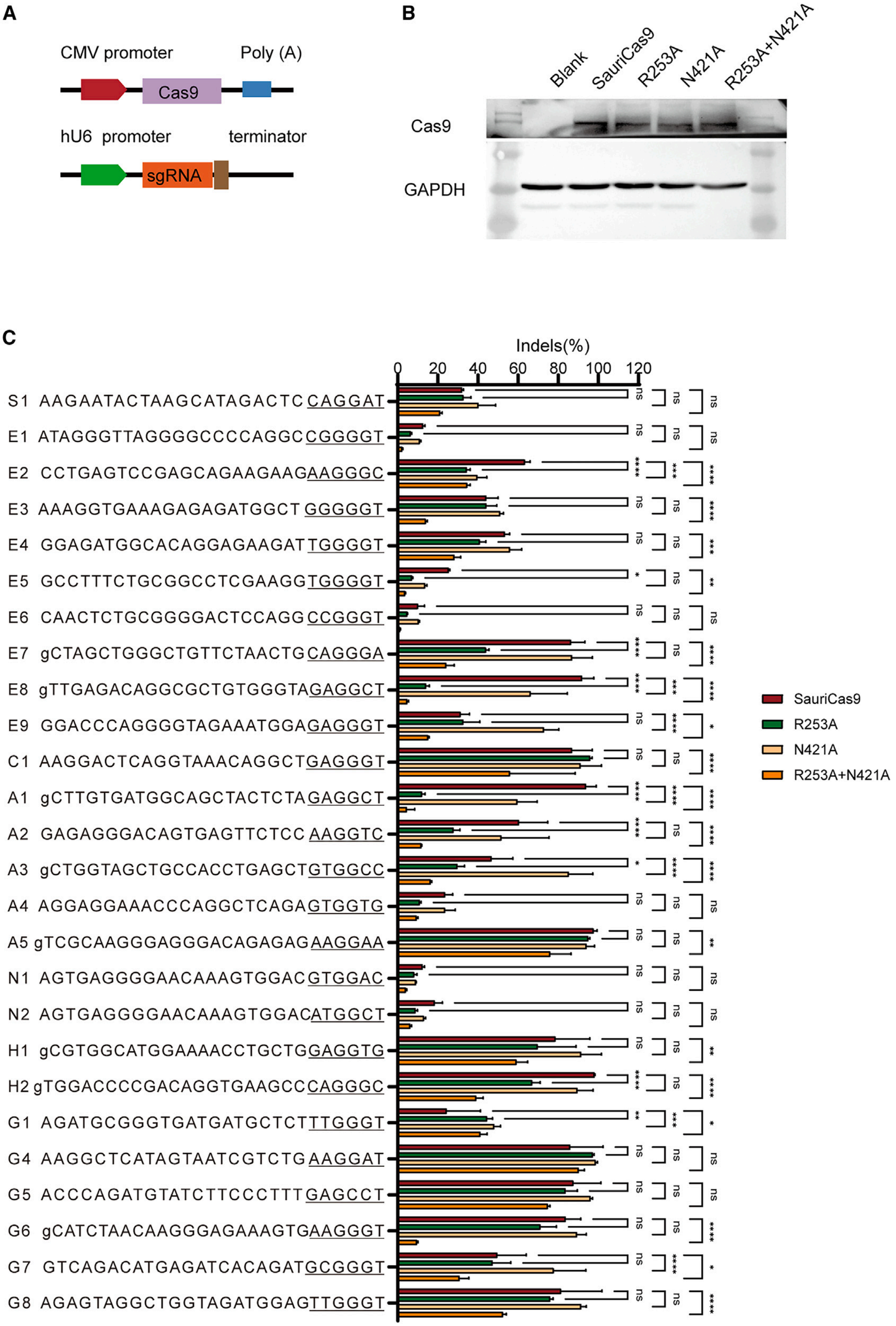

(legend on next page)

R253A + N421A exhibited the detection of eight, three, and one off-target sites, respectively. Notably, no off-target sites were identified for R253A. In summary, these findings strongly suggest that the mutations R253A, N421A, and R253A + N421A contribute to the enhanced specificity of SauriCas9.

Next, we evaluated the activity of SauriCas9, R253A, N421A, and R253A + N421A across a panel of 26 endogenous loci. All Cas9 variants and sgRNAs were expressed using identical constructs, and comparable protein expression levels were confirmed by western blot analysis (Figures 3A and 3B). Five days post-transfection of Cas9 and sgRNA-expressing plasmids into HEK293T cells, genomic DNA was extracted for targeted deep sequencing. The results revealed variable editing efficiencies depending on the targeted sites (Figure 3C). Specifically, high indel efficiencies (>30%) were observed at sites E2, E4, C1, A5, H1, H2, G4, G5, G7, and G8, while low indel efficiencies (<30%) were observed at the remaining sites. The activity of SauriCas9 was dramatically impacted by the double mutations, resulting in very low efficiencies (<5%) at sites E1, E5, E6, E8, N1, and A1. In contrast, both SauriCas9 and N421A exhibited indel efficiencies exceeding 5% at all sites, and R253A achieved indel efficiencies over 5% at 25 sites. Overall, the ranking of editing efficiencies was SauriCas9 > N421A > R253A > R253A + N421A. In addition, we compared the activity of R253A with that of two previously reported high-fidelity variants, N269D and D270N,[28] across a panel of 16 endogenous loci (Figure S2). The results showed variable editing efficiencies depending on the targeted sites. Among the 16 loci, 10 exhibited no significant difference in editing efficiency between R253A and N269D. Similarly, eight loci showed no significant difference between R253A and D270N. However, three sites (H1, G7, G8) displayed significantly lower editing efficiencies for N269D and D270N compared to R253A.

Next, we tested whether SauriCas9-R253A could be employed for base editing. To do this, we generated the nickase form of SauriCas9-R253A (SauriCas9n-R253A) by introducing the D15A mutation. We then replaced SpCas9 with SauriCas9n-R253A in ABEmax to create an adenine base editor, which we named Sauri-R253A-ABEmax (Figure S3A). We transfected HEK293T cells with plasmids encoding Sauri-R253A-ABEmax and sgRNAs targeting seven human genomic loci (Figure S3B). After 7 days of editing, targeted deep sequencing revealed successful A-to-G base editing, with an editing efficiency reaching 40.7% (Figure S3C). These results demonstrate that SauriCas9-R253A can be effectively utilized for base editing.

Next, we assessed the knockout capability of SauriCas9-R253A targeting the *B2M*, *TRAC*, and *PDCD1* genes in HEK293T cells, using SpCas9 as a control. These genes play a pivotal role in the development of universal chimeric antigen receptor T (CAR-T) cells.[29] Specifically, targeting *B2M* and *TRAC* aims to mitigate the risk of graft-versus-host disease by reducing the immune response when employing CAR-T cells from a healthy donor for patient treatment.[30] Additionally, the exploration of *PDCD1* gene knockout is aimed at potentially enhancing the anti-tumor responses of CAR-T cells.[31] For each gene, we designed five to six sgRNAs for SauriCas9-R253A (Figure 4A). In addition, we selected three to four sgRNAs from the literature for SpCas9.[29] While SauriCas9-R253A sgRNAs generally exhibited lower activity than SpCas9 sgRNAs, the most efficient SauriCas9-R253A sgRNAs (Sa-sg3 for *B2M*, Sa-sg1 for *TRAC*, and Sa-sg3 for *PDCD1*) displayed comparable efficiency to the SpCas9 sgRNAs (Figure 4B).

Finally, we evaluated the specificity of SauriCas9-R253A and SpCas9 in knocking out *B2M*, *TRAC*, and *PDCD1* genes using the GUIDE-seq assay. We selected three sgRNAs for both Cas9 nucleases. SpCas9 induced two off-targets on the *B2M* gene, two off-targets on the *PDCD1* gene, and no off-targets on the *TRAC* gene. In contrast, we did not detect off-targets for SauriCas9-R253A (Figure 4C). These data underscore the safety and precision of SauriCas9-R253A in genome editing applications.

DISCUSSION

SauriCas9, a compact Cas9 nuclease recognizing a straightforward NNGG PAM,[12] has demonstrated promising outcomes in preclinical evaluations. It efficiently edits the aberrant donor splice site in the *LZTR1* gene, resulting in the restoration of LZTR1 expression in cardiomyocytes derived from patients with Noonan syndrome.[32] Furthermore, the SauriCas9-based adenine base editor (SauriABE) showcases considerable potential for *in vivo* genome editing. When packaged into a single AAV, SauriABE efficiently disrupts Psck9 expression in the mouse liver, leading to a reduction in plasma cholesterol and triglyceride levels.[33] Notably, SauriABE also demonstrates the capability to correct an LMNA mutation associated with dilated cardiomyopathy in mouse hearts.[34]

While SauriCas9 holds promise, concerns about off-target effects have been acknowledged. In this study, we sought to enhance the safety profile of SauriCas9 by introducing mutations designed to eliminate polar contacts between Cas9 and the target DNA. Intriguingly, SauriCas9-R253A emerged as a variant with notably high specificity. Upon assessing its functionality in disrupting B2M, TRAC, and PDCD1 expression, SauriCas9-R253A exhibited comparable activity to SpCas9, particularly when optimized sgRNA designs were employed. Importantly, the application of three selected sgRNAs revealed no detectable

Figure 3. Analysis of activity for each Cas9 variant

(A) Schematic representation of the Cas9 expression constructs, where Cas9 and sgRNA are expressed on separate plasmids. (B) Measurement of Cas9 protein expression levels via western blot. "Blank" denotes cells without Cas9 transfection. (C) Evaluation of the activity of four Cas9 variants at 26 endogenous loci. For sgRNAs initiated with "C" or "T," an additional "g" is included for U6 promoter transcription ($n = 3$). The data are presented as mean ± SD. Two-tailed, paired Student's t tests were utilized to determine statistical significance when comparing two groups, whereas ANOVAs were employed for comparisons involving three or more groups. A value of $p < 0.05$ was deemed statistically significant (*$p < 0.05$; **$p < 0.01$; ***$p < 0.001$).

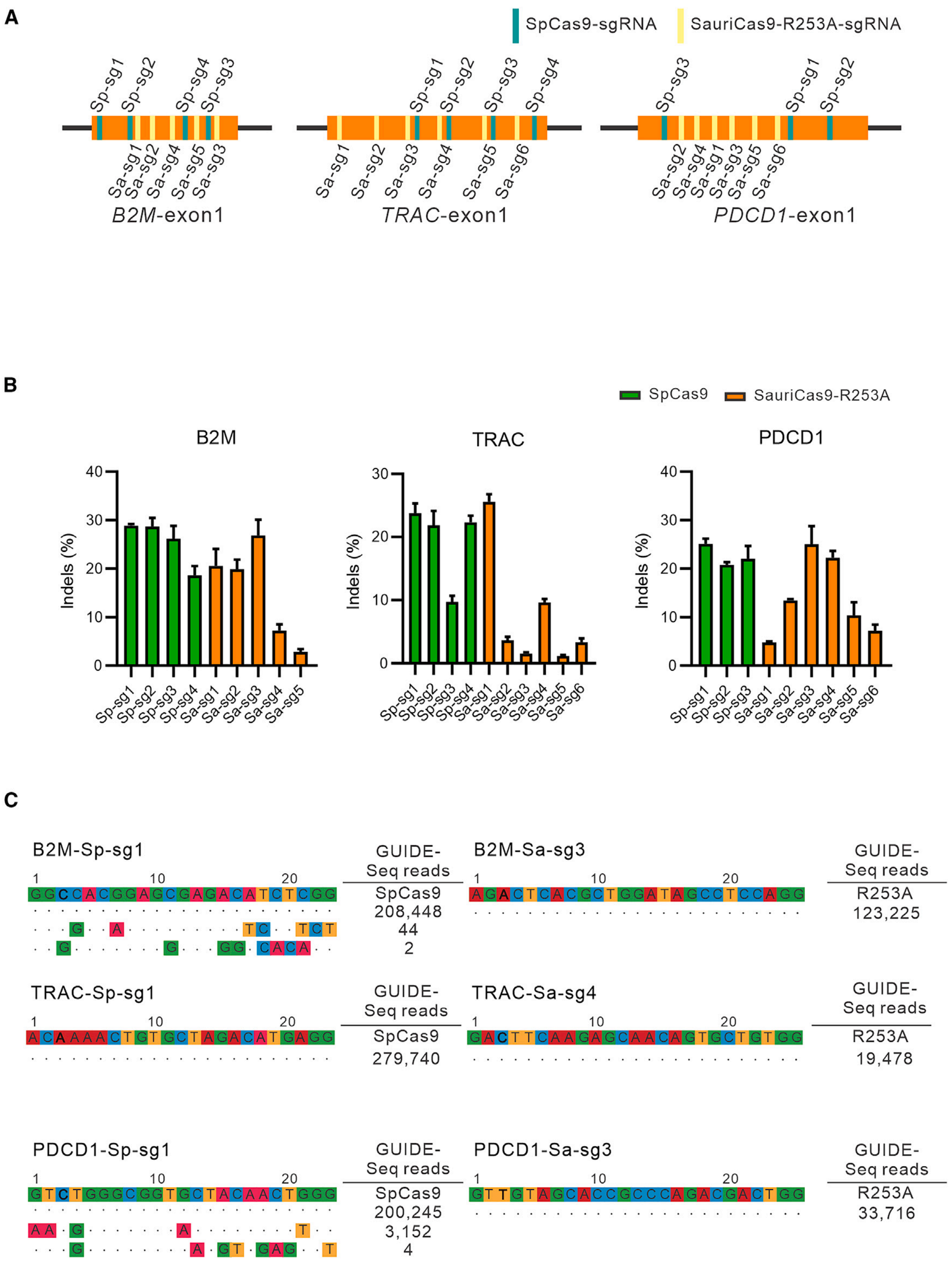

Figure 4. Genome editing of *B2M*, *TRAC*, and *PDCD1* genes with SauriCas9-R253A

(A) Schematic representation of sgRNA design targeting exon 1 of *B2M*, *TRAC*, and *PDCD1*. (B) Evaluation of genome editing efficiency for each sgRNA. (C) GUIDE-seq analysis of genome-wide off-target effects for three sgRNAs targeting the *B2M*, *TRAC*, and *PDCD1* genes. The on-target sequences are presented at the top, while mismatches compared with the on-target sequence are depicted below. The read numbers for both on- and off-targets are provided at right. The GUIDE-seq data are accessible on NCBI (BioProject: PRJNA1187307).

off-target effects, underscoring the safety credentials of SauriCas9-R253A. In addition to SauriCas9-R253A, a previous study identified two amino acid mutations, N269D and D270N, that can improve the specificity of SauriCas9.[28] These discoveries further augment the suitability of SauriCas9 for therapeutic applications.

MATERIALS AND METHODS

Plasmid construction

The plasmid pAAV-CMV-SauriCas9-puro (Addgene plasmid no. 135965) served as the platform for expressing both the wild-type SauriCas9 and various mutated versions. Mutant SauriCas9 expression plasmids listed in Table S1 were created through targeted mutagenesis, with pAAV-CMV-SauriCas9-puro as the template. The specific sgRNA target locations can be found in Table S3. The sgRNA expression plasmids for SauriCas9 were generated by inserting the sgRNA into the BsaI-digested hU6-Sa_tracr plasmid. The primer sequences are listed in Table S2. The sequence of each Cas9 construct was validated through Sanger sequencing at GENEWIZ in Suzhou, China. Subsequently, all plasmids underwent purification using the TIANprep Mini Plasmid Kit from TIANGEN and were quantified using NanoDrop 2000.

Cell culture and transfection

Culturing of HEK293T cells involved using DMEM (Gibco) with 10% fetal bovine serum (Gibco) and 1× penicillin-streptomycin (Gibco), grown at 37°C with 5% CO_2. The cells were then transfected with Lipofectamine 2000 (Life Technologies) following the provided instructions. In the context of Cas9 genome editing comparisons, 10^5 cells were transfected with a total of 300 ng Cas9 plasmid and 200 ng sgRNA plasmid in 48-well plates.

Test of Cas9 specificity

To determine the specificity of Cas9, we created one GFP reporter cell line with the CTGG PAM. Subsequently, the cells were distributed into 48-well plates and then transfected with 300 ng Cas9 plasmids and 200 ng sgRNA plasmids using Lipofectamine 2000. After 5 days of editing, the GFP^+ cells were examined using a Calibur instrument (BD Biosciences), and the resulting data were analyzed with FlowJo.

PAM library screening experiments

The PAM library cells were plated into a 10-cm dish at ∼25% confluence. At 24 h later, the PAM library cells were transfected with Cas9-gRNA expressing plasmid (10 μg). Five days after editing, the GFP^+ cells were sorted by the MoFlo XDP machine, and the genomic DNA was isolated using the TIANamp Genomic DNA Kit (TIANGEN) following the manufacturer's instructions. PCR fragments for deep sequencing were generated in two-step PCR reactions. First, the target region was PCR amplified using primers Deep-F1/R1 with 25 cycles using Q5 High-Fidelity 2× Master Mix (NEB). Second, 3 μL PCR products from the first step was amplified by primer P5-adapter-F and P7-adapter-R for 15 cycles (Table S2). The PCR products were purified using the Qiagen Gel Extraction Kit and were sequenced on Illumina HiSeq X by 150-bp paired-end sequencing.

PAM sequence analysis

Twenty base pair sequences (5′-AAGCCTTGTTTGCCACCATG/GTGAGCAAGGGCG AGGAGCT-3′) flanking the target sequence (5′-GAACGGCTCGGAGATCATCATTGCG NNNNNNN-3′) were used to fix the target sequence. 5′-GCG-3′ and 5′-GTGAGCAAG GGCG AGGAGCT-3′ were used to fix the 7-bp random sequence. Target sequences with in-frame mutations were used for PAM analysis. The 7-bp random sequence was extracted and visualized by WebLog3.[35]

GUIDE-seq

GUIDE-seq experiments were conducted following previously established procedures with slight modifications. In brief, 2 × 10^5 HEK293T cells were transfected with 500 ng Cas9, 500 ng sgRNA plasmids, and 100 pmol annealed GUIDE-seq oligonucleotides via electroporation and subsequently distributed into 6-well plates. The electroporation settings used were 1,150 V for voltage, 30 ms for width, and 1 pulse for the number of pulses. Genomic DNA was extracted 6 days post-transfection using the DNeasy Blood and Tissue kit (Qiagen) in accordance with the manufacturer's protocol, followed by preparation of the genome library for deep sequencing.

Genome editing for endogenous sites

The experimental procedure involved seeding HEK293T cells into 48-well plates, followed by transfection with a combined total of 300 ng Cas9 plasmid and 200 ng sgRNA plasmid using Lipofectamine 2000. Cells were harvested after a 5-day incubation post-transfection. Subsequently, genomic DNA was extracted, and the target sites were PCR amplified and isolated using QuickExtract DNA Extraction Solution (Epicentre) for deep sequencing analysis. Detailed primer sequences can be found in Table S2.

Base editing with Sauri-R253A-ABEmax

HEK293T cells were seeded into 48-well plates and transfected with Sauri-R253A-ABEmax and hU6-Sa_tracr-gRNA. Cells were collected, and the genomic DNA was isolated 7 days after transfection. The target sites were PCR amplified and extracted by a Qiagen Gel Extraction Kit. The efficiency of the base editing was measured by deep sequencing.

Western blotting

On day 0, HEK293T cells were plated in a 6-well dish. On day 1, each well received 2 μg Cas9-expressing plasmid, which was transfected using 4 μL Lipofectamine 2000. Three days post-transfection, cell samples were collected and total proteins were extracted using NP-40 buffer (Beyotime) supplemented with 1 mM phenylmethanesulfonyl fluoride (Beyotime). The proteins were then separated by SDS-PAGE and transferred onto a polyvinylidene fluoride membrane (Thermo Fisher). Following the transfer, the membrane was blocked with 5% (w/v) BSA (Sigma) in 0.1% Tween 20 in 1× Tris-buffered saline (TBS-T) buffer. Membranes were blotted with antibodies directed at the following proteins: anti-hemagglutinin tag (1:1,000; ab236632, Abcam) and anti-GAPDH (1:2,000; 5174s, Cell Signaling Technology) at 4°C overnight. The membrane was washed three times in

TBS-T for 5 min each. Subsequently, the secondary antibody (1:10,000; ab6721, Abcam) was incubated for 1 h at room temperature, followed by three washes. Finally, protein expression levels were quantified using ImageJ.

Statistical analysis

The data are presented as mean ± SD. Statistical analyses were conducted using GraphPad Prism. Two-tailed, paired Student's t tests were utilized to determine statistical significance when comparing two groups, whereas analyses of variance (ANOVAs) were employed for comparisons involving three or more groups. A value of $p < 0.05$ was deemed statistically significant (*$p < 0.05$; **$p < 0.01$; ***$p < 0.001$).

DATA AND CODE AVAILABILITY

The authors confirm that the data supporting the findings of this study are available within the article and its supplemental information. Additional details will be made available upon request by the corresponding author, Yongming Wang.

ACKNOWLEDGMENTS

This work was supported by grants from the National Key R&D Program of China (2021YFD1400300), the National Key Research and Development Program of China (2021YFC2701103 and 2021YFA0910602), the Scientific Research Foundation provided by Pudong Hospital affiliated to Fudan University (YJYJRC202308), the National Natural Science Foundation of China (82070258 and 82370254), and the Science and Technology Research Program of Shanghai (24HC2810100 and 23ZR1426000).

AUTHOR CONTRIBUTIONS

X.Z., C.T., and M.L. performed the experiments; H.L., Y.W., S.Z., Y.H., and P.L. provided experimental guidance; Y.W. wrote the manuscript.

DECLARATION OF INTERESTS

The authors declare no competing interests.

SUPPLEMENTAL INFORMATION

Supplemental information can be found online at https://doi.org/10.1016/j.omtn.2025.102455.

REFERENCES

1. Jinek, M., Chylinski, K., Fonfara, I., Hauer, M., Doudna, J.A., and Charpentier, E. (2012). A programmable dual-RNA-guided DNA endonuclease in adaptive bacterial immunity. Science *337*, 816–821. https://doi.org/10.1126/science.1225829.

2. Nishimasu, H., Cong, L., Yan, W.X., Ran, F.A., Zetsche, B., Li, Y., Kurabayashi, A., Ishitani, R., Zhang, F., and Nureki, O. (2015). Crystal Structure of Staphylococcus aureus Cas9. Cell *162*, 1113–1126. https://doi.org/10.1016/j.cell.2015.08.007.

3. Ran, F.A., Hsu, P.D., Wright, J., Agarwala, V., Scott, D.A., and Zhang, F. (2013). Genome engineering using the CRISPR-Cas9 system. Nat. Protoc. *8*, 2281–2308. https://doi.org/10.1038/nprot.2013.143.

4. Komor, A.C., Kim, Y.B., Packer, M.S., Zuris, J.A., and Liu, D.R. (2016). Programmable editing of a target base in genomic DNA without double-stranded DNA cleavage. Nature *533*, 420–424. https://doi.org/10.1038/nature17946.

5. Gaudelli, N.M., Komor, A.C., Rees, H.A., Packer, M.S., Badran, A.H., Bryson, D.I., and Liu, D.R. (2017). Programmable base editing of A*T to G*C in genomic DNA without DNA cleavage. Nature *551*, 464–471. https://doi.org/10.1038/nature24644.

6. Anzalone, A.V., Randolph, P.B., Davis, J.R., Sousa, A.A., Koblan, L.W., Levy, J.M., Chen, P.J., Wilson, C., Newby, G.A., Raguram, A., and Liu, D.R. (2019). Search-and-replace genome editing without double-strand breaks or donor DNA. Nature *576*, 149–157. https://doi.org/10.1038/s41586-019-1711-4.

7. Cong, L., Ran, F.A., Cox, D., Lin, S., Barretto, R., Habib, N., Hsu, P.D., Wu, X., Jiang, W., Marraffini, L.A., and Zhang, F. (2013). Multiplex genome engineering using CRISPR/Cas systems. Science *339*, 819–823. https://doi.org/10.1126/science.1231143.

8. Mali, P., Yang, L., Esvelt, K.M., Aach, J., Guell, M., DiCarlo, J.E., Norville, J.E., and Church, G.M. (2013). RNA-guided human genome engineering via Cas9. Science *339*, 823–826. https://doi.org/10.1126/science.1232033.

9. Ran, F.A., Cong, L., Yan, W.X., Scott, D.A., Gootenberg, J.S., Kriz, A.J., Zetsche, B., Shalem, O., Wu, X., Makarova, K.S., et al. (2015). In vivo genome editing using Staphylococcus aureus Cas9. Nature *520*, 186–191. https://doi.org/10.1038/nature14299.

10. Edraki, A., Mir, A., Ibraheim, R., Gainetdinov, I., Yoon, Y., Song, C.Q., Cao, Y., Gallant, J., Xue, W., Rivera-Pérez, J.A., and Sontheimer, E.J. (2019). A Compact, High-Accuracy Cas9 with a Dinucleotide PAM for In Vivo Genome Editing. Mol. Cell *73*, 714–726.e4. https://doi.org/10.1016/j.molcel.2018.12.003.

11. Kim, E., Koo, T., Park, S.W., Kim, D., Kim, K., Cho, H.Y., Song, D.W., Lee, K.J., Jung, M.H., Kim, S., et al. (2017). In vivo genome editing with a small Cas9 orthologue derived from Campylobacter jejuni. Nat. Commun. *8*, 14500. https://doi.org/10.1038/ncomms14500.

12. Hu, Z., Wang, S., Zhang, C., Gao, N., Li, M., Wang, D., Wang, D., Liu, D., Liu, H., Ong, S.G., et al. (2020). A compact Cas9 ortholog from Staphylococcus Auricularis (SauriCas9) expands the DNA targeting scope. PLoS Biol. *18*, e3000686. https://doi.org/10.1371/journal.pbio.3000686.

13. Hu, Z., Zhang, C., Wang, S., Gao, S., Wei, J., Li, M., Hou, L., Mao, H., Wei, Y., Qi, T., et al. (2021). Discovery and engineering of small SlugCas9 with broad targeting range and high specificity and activity. Nucleic Acids Res. *49*, 4008–4019. https://doi.org/10.1093/nar/gkab148.

14. Wang, S., Mao, H., Hou, L., Hu, Z., Wang, Y., Qi, T., Tao, C., Yang, Y., Zhang, C., Li, M., et al. (2022). Compact SchCas9 Recognizes the Simple NNGR PAM. Adv. Sci. *9*, 2104789.

15. Wei, J., Hou, L., Liu, J., Wang, Z., Gao, S., Qi, T., Gao, S., Sun, S., and Wang, Y. (2022). Closely related type II-C Cas9 orthologs recognize diverse PAMs. Elife *11*, e77825.

16. Wang, S., Tao, C., Mao, H., Hou, L., Wang, Y., Qi, T., Yang, Y., Ong, S.G., Hu, S., Chai, R., and Wang, Y. (2022). Identification of SaCas9 orthologs containing a conserved serine residue that determines simple NNGG PAM recognition. PLoS Biol. *20*, e3001897.

17. Gao, S., Wang, Y., Qi, T., Wei, J., Hu, Z., Liu, J., Sun, S., Liu, H., and Wang, Y. (2023). Genome editing with natural and engineered CjCas9 orthologs. Mol. Ther. *31*, 1177–1187. https://doi.org/10.1016/j.ymthe.2023.01.029.

18. Liu, J.J., Orlova, N., Oakes, B.L., Ma, E., Spinner, H.B., Baney, K.L.M., Chuck, J., Tan, D., Knott, G.J., Harrington, L.B., et al. (2019). CasX enzymes comprise a distinct family of RNA-guided genome editors. Nature *566*, 218–223. https://doi.org/10.1038/s41586-019-0908-x.

19. Kim, D.Y., Lee, J.M., Moon, S.B., Chin, H.J., Park, S., Lim, Y., Kim, D., Koo, T., Ko, J.H., and Kim, Y.S. (2022). Efficient CRISPR editing with a hypercompact Cas12f1 and engineered guide RNAs delivered by adeno-associated virus. Nat. Biotechnol. *40*, 94–102. https://doi.org/10.1038/s41587-021-01009-z.

20. Xu, X., Chemparathy, A., Zeng, L., Kempton, H.R., Shang, S., Nakamura, M., and Qi, L.S. (2021). Engineered miniature CRISPR-Cas system for mammalian genome regulation and editing. Mol. Cell *81*, 4333–4345.e4. https://doi.org/10.1016/j.molcel.2021.08.008.

21. Wu, Z., Zhang, Y., Yu, H., Pan, D., Wang, Y., Wang, Y., Li, F., Liu, C., Nan, H., Chen, W., and Ji, Q. (2021). Programmed genome editing by a miniature CRISPR-Cas12f nuclease. Nat. Chem. Biol. *17*, 1132–1138. https://doi.org/10.1038/s41589-021-00868-6.

22. Pausch, P., Al-Shayeb, B., Bisom-Rapp, E., Tsuchida, C.A., Li, Z., Cress, B.F., Knott, G.J., Jacobsen, S.E., Banfield, J.F., and Doudna, J.A. (2020). CRISPR-Cas Phi from huge phages is a hypercompact genome editor. Science *369*, 333–337. https://doi.org/10.1126/science.abb1400.

23. Wang, Y., Qi, T., Liu, J., Yang, Y., Wang, Z., Wang, Y., Wang, T., Li, M., Li, M., Lu, D., et al. (2023). A highly specific CRISPR-Cas12j nuclease enables allele- specific genome editing. Sci. Adv. *9*, eabo6405.

24. Slaymaker, I.M., Gao, L., Zetsche, B., Scott, D.A., Yan, W.X., and Zhang, F. (2016). Rationally engineered Cas9 nucleases with improved specificity. Science *351*, 84–88. https://doi.org/10.1126/science.aad5227.

25. Tan, Y., Chu, A.H.Y., Bao, S., Hoang, D.A., Kebede, F.T., Xiong, W., Ji, M., Shi, J., and Zheng, Z. (2019). Rationally engineered Staphylococcus aureus Cas9 nucleases with high genome-wide specificity. Proc. Natl. Acad. Sci. USA *116*, 20969–20976. https://doi.org/10.1073/pnas.1906843116.

26. Lee, J.K., Jeong, E., Lee, J., Jung, M., Shin, E., Kim, Y.H., Lee, K., Jung, I., Kim, D., Kim, S., and Kim, J.S. (2018). Directed evolution of CRISPR-Cas9 to increase its specificity. Nat. Commun. *9*, 3048.

27. Tsai, S.Q., Zheng, Z., Nguyen, N.T., Liebers, M., Topkar, V.V., Thapar, V., Wyvekens, N., Khayter, C., Iafrate, A.J., Le, L.P., et al. (2015). GUIDE-seq enables genome-wide profiling of off-target cleavage by CRISPR-Cas nucleases. Nat. Biotechnol. *33*, 187–197. https://doi.org/10.1038/nbt.3117.

28. Wei, N., Shang, L., Liu, J., Wang, M., Liu, Y., Zhu, C., Fei, C., Zhang, L., Yang, F., and Gu, F. (2023). Engineered Staphylococcus auricularis Cas9 with high-fidelity. FASEB J. *37*, e23060. https://doi.org/10.1096/fj.202202132RR.

29. Liu, X., Zhang, Y., Cheng, C., Cheng, A.W., Zhang, X., Li, N., Xia, C., Wei, X., Liu, X., and Wang, H. (2017). CRISPR-Cas9-mediated multiplex gene editing in CAR-T cells. Cell Res. *27*, 154–157. https://doi.org/10.1038/cr.2016.142.

30. Riolobos, L., Hirata, R.K., Turtle, C.J., Wang, P.R., Gornalusse, G.G., Zavajlevski, M., Riddell, S.R., and Russell, D.W. (2013). HLA engineering of human pluripotent stem cells. Mol. Ther. *21*, 1232–1241. https://doi.org/10.1038/mt.2013.59.

31. Kamphorst, A.O., and Ahmed, R. (2013). Manipulating the PD-1 pathway to improve immunity. Curr. Opin. Immunol. *25*, 381–388. https://doi.org/10.1016/j.coi.2013.03.003.

32. Knauer, C., Haltern, H., Schoger, E., Kügler, S., Roos, L., Zelarayán, L.C., Hasenfuss, G., Zimmermann, W.H., Wollnik, B., and Cyganek, L. (2024). Preclinical evaluation of CRISPR-based therapies for Noonan syndrome caused by deep-intronic LZTR1 variants. Mol. Ther. Nucleic Acids *35*, 102123. https://doi.org/10.1016/j.omtn.2024.102123.

33. Davis, J.R., Wang, X., Witte, I.P., Huang, T.P., Levy, J.M., Raguram, A., Banskota, S., Seidah, N.G., Musunuru, K., and Liu, D.R. (2022). Efficient in vivo base editing via single adeno-associated viruses with size-optimized genomes encoding compact adenine base editors. Nat. Biomed. Eng. *6*, 1272–1283. https://doi.org/10.1038/s41551-022-00911-4.

34. Yang, L., Liu, Z., Sun, J., Chen, Z., Gao, F., and Guo, Y. (2024). Adenine base editor-based correction of the cardiac pathogenic Lmna c.1621C > T mutation in murine hearts. J. Cell Mol. Med. *28*, e18145. https://doi.org/10.1111/jcmm.18145.

35. Crooks, G.E., Hon, G., Chandonia, J.M., and Brenner, S.E. (2004). WebLogo: a sequence logo generator. Genome Res. *14*, 1188–1190. https://doi.org/10.1101/gr.849004.

OMTN, Volume *36*

Supplemental information

Engineering of SauriCas9

with enhanced specificity

Xiaoqi Zhang, Chen Tao, Miaomiao Li, Sufang Zhang, Puping Liang, Yan Huang, Huihui Liu, and Yongming Wang

TableS1 SauriCas9 and SauriCas9 variants

Table S2. Primers used in this study

Table S3. Target sites used in this study

Table S4. Data in this study

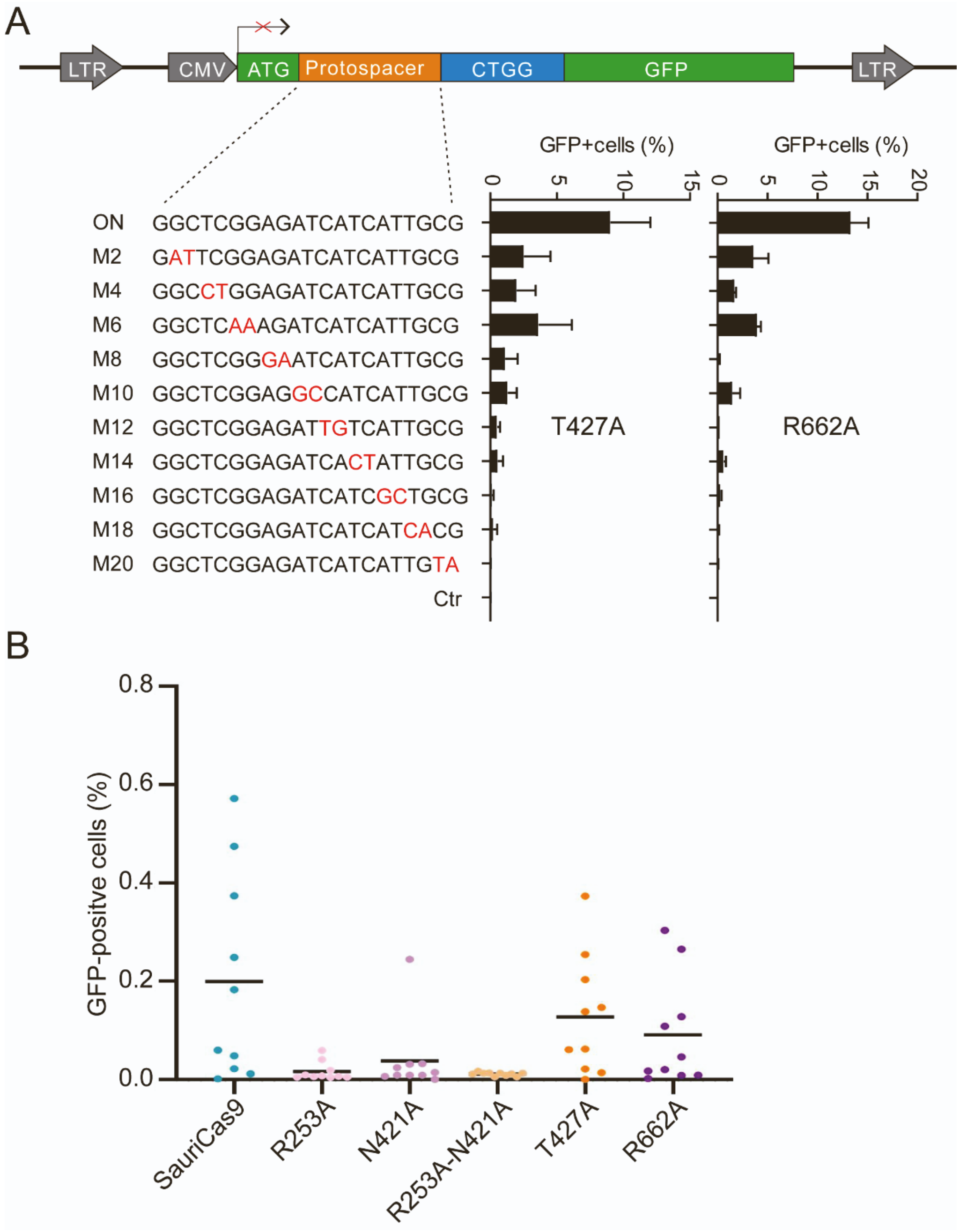

Fig. S1 Influence of mutations on SauriCas9 specificity. (A) Evaluation of Cas9 specificity using the GFP-activation assay. The top section illustrates the schematic of the GFP-activation reporter. A series of sgRNAs with dinucleotide mutations (depicted in red) is displayed below. The percentage of GFP-positive cells for each sgRNA is indicated on the right. The on-target sgRNA is included for reference. Ctr: reporter cells without Cas9-expressing plasmid transfection. **(B)** Comparison of editing efficiency at off-targets based on GFP-activation assay among six SauriCas9 variants in HEK293T cells. The editing efficiency at off-targets is normalized by on-target efficiency. Low editing efficiency indicates high specificity.

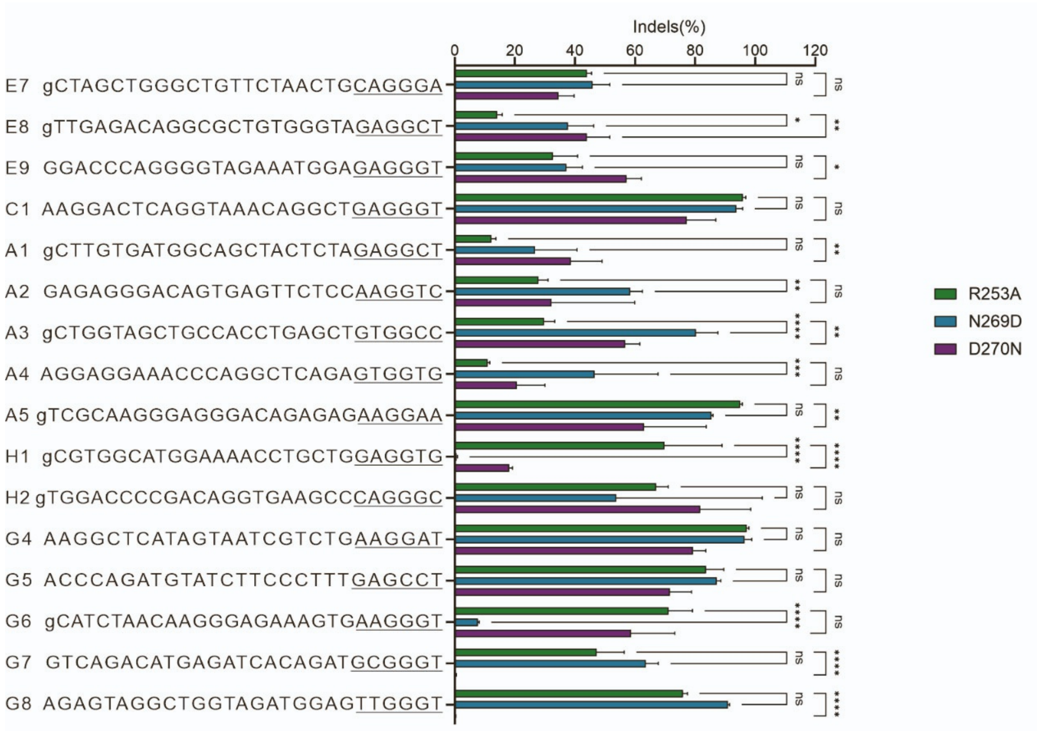

Fig. S2 Evaluation of the activity of three Cas9 variants at sixteen endogenous loci. The data is presented as mean ± SD (n = 3). Two-tailed, paired Student t-tests were utilized to determine statistical significance when comparing two groups, whereas analyses of variance (ANOVAs) were employed for comparisons involving three or more groups. A value of $P < 0.05$ was deemed statistically significant (*$P < 0.05$, **$P < 0.01$, ***$P < 0.001$).

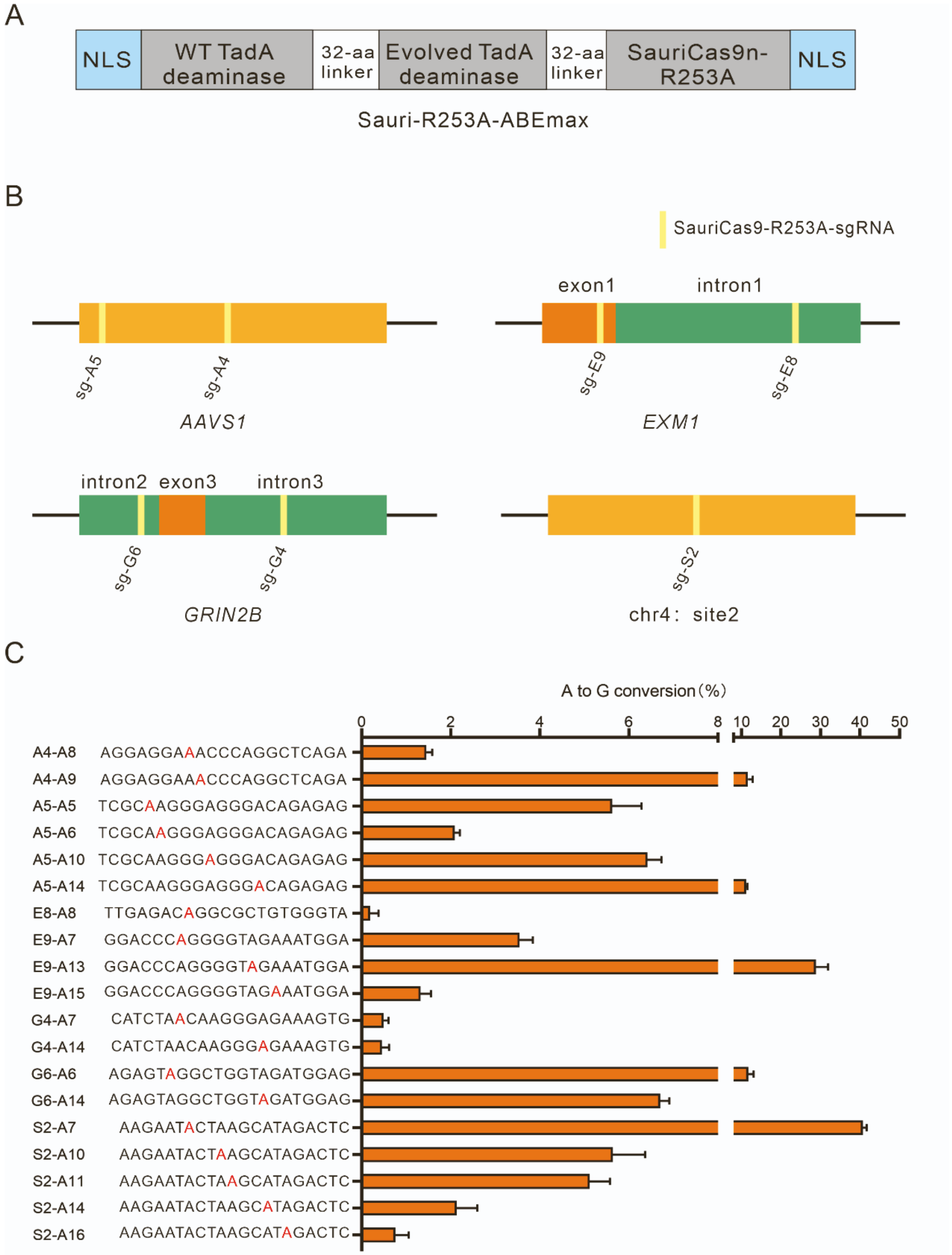

Fig. S3 Base editing with SauriCas9-R253A. (A) Schematic of the Sauri-R253A-ABEmax. **(B)** Schematic representation of sgRNA design targeting AAVS1, EXM1, GRIN2B and site2. Yellow boxes indicate the sgRNA targeting sites. **(C)** Sauri-R253A-ABEmax induces A-to-G conversions for a panel of 7 genomic loci (*n* = 3). Underlying data for all summary statistics can be found in Data. “A4-A8” means A at target A4 position 8. aa, amino acid; NLS, nuclear localization signal; Sauri-R253A-ABEmax, TadA-TadA*(involved TadA)–SauriCas9n-R253A; SauriCas9, Cas9 nuclease from *S. auricularis*; SauriCas9n, nickase form of SauriCas9.

References

1. Jinek, M., Chylinski, K., Fonfara, I., Hauer, M., Doudna, J.A., and Charpentier, E. (2012). A programmable dual-RNA-guided DNA endonuclease in adaptive bacterial immunity. Science *337*, 816-821. 10.1126/science.1225829.
2. Nishimasu, H., Cong, L., Yan, W.X., Ran, F.A., Zetsche, B., Li, Y., Kurabayashi, A., Ishitani, R., Zhang, F., and Nureki, O. (2015). Crystal Structure of Staphylococcus aureus Cas9. Cell *162*, 1113-1126. 10.1016/j.cell.2015.08.007.
3. Ran, F.A., Hsu, P.D., Wright, J., Agarwala, V., Scott, D.A., and Zhang, F. (2013). Genome engineering using the CRISPR-Cas9 system. Nature Protocols *8*, 2281-2308. 10.1038/nprot.2013.143.
4. Komor, A.C., Kim, Y.B., Packer, M.S., Zuris, J.A., and Liu, D.R. (2016). Programmable editing of a target base in genomic DNA without double-stranded DNA cleavage. Nature *533*, 420-424. 10.1038/nature17946.
5. Gaudelli, N.M., Komor, A.C., Rees, H.A., Packer, M.S., Badran, A.H., Bryson, D.I., and Liu, D.R. (2017). Programmable base editing of A*T to G*C in genomic DNA without DNA cleavage. Nature *551*, 464-471. 10.1038/nature24644.
6. Anzalone, A.V., Randolph, P.B., Davis, J.R., Sousa, A.A., Koblan, L.W., Levy, J.M., Chen, P.J., Wilson, C., Newby, G.A., Raguram, A., and Liu, D.R. (2019). Search-and-replace genome editing without double-strand breaks or donor DNA. Nature *576*, 149-157. 10.1038/s41586-019-1711-4.
7. Cong, L., Ran, F.A., Cox, D., Lin, S., Barretto, R., Habib, N., Hsu, P.D., Wu, X., Jiang, W., Marraffini, L.A., and Zhang, F. (2013). Multiplex genome engineering using CRISPR/Cas systems. Science *339*, 819-823. 10.1126/science.1231143.
8. Mali, P., Yang, L., Esvelt, K.M., Aach, J., Guell, M., DiCarlo, J.E., Norville, J.E., and Church, G.M. (2013). RNA-guided human genome engineering via Cas9. Science *339*, 823-826. 10.1126/science.1232033.
9. Ran, F.A., Cong, L., Yan, W.X., Scott, D.A., Gootenberg, J.S., Kriz, A.J., Zetsche, B., Shalem, O., Wu, X., Makarova, K.S., et al. (2015). In vivo genome editing using Staphylococcus aureus Cas9. Nature *520*, 186-191. 10.1038/nature14299.
10. Edraki, A., Mir, A., Ibraheim, R., Gainetdinov, I., Yoon, Y., Song, C.Q., Cao, Y., Gallant, J., Xue, W., Rivera-Perez, J.A., and Sontheimer, E.J. (2019). A Compact, High-Accuracy Cas9 with a Dinucleotide PAM for In Vivo Genome Editing. Mol Cell *73*, 714-726 e714. 10.1016/j.molcel.2018.12.003.
11. Kim, E., Koo, T., Park, S.W., Kim, D., Kim, K., Cho, H.Y., Song, D.W., Lee, K.J., Jung, M.H., Kim, S., et al. (2017). In vivo genome editing with a small Cas9 orthologue derived from Campylobacter jejuni. Nat Commun *8*, 14500. 10.1038/ncomms14500.
12. Hu, Z., Wang, S., Zhang, C., Gao, N., Li, M., Wang, D., Wang, D., Liu, D., Liu, H., Ong, S.G., et al. (2020). A compact Cas9 ortholog from Staphylococcus Auricularis (SauriCas9) expands the DNA targeting scope. PLoS Biol *18*, e3000686. 10.1371/journal.pbio.3000686.
13. Hu, Z., Zhang, C., Wang, S., Gao, S., Wei, J., Li, M., Hou, L., Mao, H., Wei, Y., Qi, T., et al. (2021). Discovery and engineering of small SlugCas9 with broad targeting range and high specificity and activity. Nucleic Acids Res *49*, 4008-4019. 10.1093/nar/gkab148.
14. Wang, S., Mao, H.L., Hou, L.H., Hu, Z.Y., Wang, Y., Qi, T., Tao, C., Yang, Y., Zhang, C.D., Li, M.M., et al. (2021). Compact SchCas9 Recognizes the Simple NNGR PAM. Advanced

Science. Artn 2104789
10.1002/Advs.202104789.
15. Wei, J.J., Hou, L.H., Liu, J.T., Wang, Z.W., Gao, S.Q., Qi, T., Gao, S., Sun, S.A., and Wang, Y.M. (2022). Closely related type II-C Cas9 orthologs recognize diverse PAMs. Elife *11*. ARTN e77825
10.7554/eLife.77825.
16. Wang, S., Tao, C., Mao, H.L., Hou, L.H., Wang, Y., Qi, T., Yang, Y., Ong, S.G., Hu, S.J., Chai, R.J., and Wang, Y.M. (2022). Identification of SaCas9 orthologs containing a conserved serine residue that determines simple NNGG PAM recognition. Plos Biology *20*. ARTN e3001897
10.1371/journal.pbio.3001897.
17. Gao, S.Q., Wang, Y., Qi, T., Wei, J.J., Hu, Z.Y., Liu, J.T., Sun, S.A., Liu, H.H., and Wang, Y.M. (2023). Genome editing with natural and engineered CjCas9 orthologs. Molecular Therapy *31*, 1177-1187. 10.1016/j.ymthe.2023.01.029.
18. Liu, J.J., Orlova, N., Oakes, B.L., Ma, E., Spinner, H.B., Baney, K.L.M., Chuck, J., Tan, D., Knott, G.J., Harrington, L.B., et al. (2019). CasX enzymes comprise a distinct family of RNA-guided genome editors. Nature *566*, 218-223. 10.1038/s41586-019-0908-x.
19. Kim, D.Y., Lee, J.M., Moon, S.B., Chin, H.J., Park, S., Lim, Y., Kim, D., Koo, T., Ko, J.H., and Kim, Y.S. (2022). Efficient CRISPR editing with a hypercompact Cas12f1 and engineered guide RNAs delivered by adeno-associated virus. Nature Biotechnology *40*, 94-+. 10.1038/s41587-021-01009-z.
20. Xu, X.S., Chemparathy, A., Zeng, L.P., Kempton, H.R., Shang, S., Nakamura, M., and Qi, L.S. (2021). Engineered miniature CRISPR-Cas system for mammalian genome regulation and editing. Molecular Cell *81*, 4333-+. 10.1016/j.molcel.2021.08.008.
21. Wu, Z.W., Zhang, Y.F., Yu, H.P., Pan, D., Wang, Y.J., Wang, Y.N., Li, F., Liu, C., Nan, H., Chen, W.Z., and Ji, Q.J. (2021). Programmed genome editing by a miniature CRISPR-Cas12f nuclease. Nature Chemical Biology *17*, 1132-1138. 10.1038/s41589-021-00868-6.
22. Pausch, P., Al-Shayeb, B., Bisom-Rapp, E., Tsuchida, C.A., Li, Z., Cress, B.F., Knott, G.J., Jacobsen, S.E., Banfield, J.F., and Doudna, J.A. (2020). CRISPR-Cas Phi from huge phages is a hypercompact genome editor. Science *369*, 333-+. 10.1126/science.abb1400.
23. Wang, Y., Qi, T., Liu, J.T., Yang, Y., Wang, Z.W., Wang, Y., Wang, T.Y., Li, M.M., Li, M.Q., Lu, D.R., et al. (2023). A highly specific CRISPR-Cas12j nuclease enables allele- specific genome editing. Science Advances *9*. ARTN eabo6405
10.1126/sciadv.abo6405.
24. Slaymaker, I.M., Gao, L., Zetsche, B., Scott, D.A., Yan, W.X., and Zhang, F. (2016). Rationally engineered Cas9 nucleases with improved specificity. Science *351*, 84-88. 10.1126/science.aad5227.
25. Tan, Y., Chu, A.H.Y., Bao, S., Hoang, D.A., Kebede, F.T., Xiong, W., Ji, M., Shi, J., and Zheng, Z. (2019). Rationally engineered Staphylococcus aureus Cas9 nucleases with high genome-wide specificity. Proc Natl Acad Sci U S A *116*, 20969-20976. 10.1073/pnas.1906843116.
26. Lee, J.K., Jeong, E., Lee, J., Jung, M., Shin, E., Kim, Y.H., Lee, K., Jung, I., Kim, D., Kim, S., and Kim, J.S. (2018). Directed evolution of CRISPR-Cas9 to increase its specificity. Nature Communications *9*. ARTN 3048

10.1038/s41467-018-05477-x.

27. Tsai, S.Q., Zheng, Z., Nguyen, N.T., Liebers, M., Topkar, V.V., Thapar, V., Wyvekens, N., Khayter, C., Iafrate, A.J., Le, L.P., et al. (2015). GUIDE-seq enables genome-wide profiling of off-target cleavage by CRISPR-Cas nucleases. Nat Biotechnol *33*, 187-197. 10.1038/nbt.3117.
28. Wei, N., Shang, L., Liu, J., Wang, M., Liu, Y., Zhu, C., Fei, C., Zhang, L., Yang, F., and Gu, F. (2023). Engineered Staphylococcus auricularis Cas9 with high-fidelity. FASEB J *37*, e23060. 10.1096/fj.202202132RR.
29. Liu, X., Zhang, Y., Cheng, C., Cheng, A.W., Zhang, X., Li, N., Xia, C., Wei, X., Liu, X., and Wang, H. (2017). CRISPR-Cas9-mediated multiplex gene editing in CAR-T cells. Cell Res *27*, 154-157. 10.1038/cr.2016.142.
30. Riolobos, L., Hirata, R.K., Turtle, C.J., Wang, P.R., Gornalusse, G.G., Zavajlevski, M., Riddell, S.R., and Russell, D.W. (2013). HLA engineering of human pluripotent stem cells. Mol Ther *21*, 1232-1241. 10.1038/mt.2013.59.
31. Kamphorst, A.O., and Ahmed, R. (2013). Manipulating the PD-1 pathway to improve immunity. Curr Opin Immunol *25*, 381-388. 10.1016/j.coi.2013.03.003.
32. Knauer, C., Haltern, H., Schoger, E., Kugler, S., Roos, L., Zelarayan, L.C., Hasenfuss, G., Zimmermann, W.H., Wollnik, B., and Cyganek, L. (2024). Preclinical evaluation of CRISPR-based therapies for Noonan syndrome caused by deep-intronic LZTR1 variants. Mol Ther Nucleic Acids *35*, 102123. 10.1016/j.omtn.2024.102123.
33. Davis, J.R., Wang, X., Witte, I.P., Huang, T.P., Levy, J.M., Raguram, A., Banskota, S., Seidah, N.G., Musunuru, K., and Liu, D.R. (2022). Efficient in vivo base editing via single adeno-associated viruses with size-optimized genomes encoding compact adenine base editors. Nat Biomed Eng *6*, 1272-1283. 10.1038/s41551-022-00911-4.
34. Yang, L., Liu, Z., Sun, J., Chen, Z., Gao, F., and Guo, Y. (2024). Adenine base editor-based correction of the cardiac pathogenic Lmna c.1621C > T mutation in murine hearts. J Cell Mol Med *28*, e18145. 10.1111/jcmm.18145.
35. Crooks, G.E., Hon, G., Chandonia, J.M., and Brenner, S.E. (2004). WebLogo: a sequence logo generator. Genome Res *14*, 1188-1190. 10.1101/gr.849004.
